# The regulatory ancestral network of surgical meshes

**DOI:** 10.1371/journal.pone.0197883

**Published:** 2018-06-19

**Authors:** Nasim Zargar, Andrew Carr

**Affiliations:** Nuffield Department of Orthopaedics, Rheumatology and Musculoskeletal Sciences (NDORMS), Botnar Research Centre, University of Oxford, Headington, Oxford, United Kingdom; Public Library of Science, UNITED KINGDOM

## Abstract

**Background:**

All surgical meshes entering the U.S. market have been cleared for clinical use by the 510(k) process of the Food and Drug Administration (FDA), in which devices simply require proof of “substantial equivalence” to predicate devices, without the need for clinical trials. However, recalled meshes associated with adverse effects may, indirectly, continue to serve as predicates for new devices raising concerns over the safety of the 510(k) route.

**Methodology:**

Here we assess the potential magnitude of this problem by determining the ancestral network of equivalence claims linking recently cleared surgical meshes. Using the FDA website we identified all surgical meshes cleared by the 510(k) route between January 2013 and December 2015 along with all listed predicates for these devices. Using a network approach, we trace the ancestry of predicates across multiple generations of equivalence claims and identify those meshes connected to devices that have since recalled from the market along with the reason for their recall.

**Conclusions:**

We find that the 77 surgical meshes cleared between 2013 and 2015 are based on 771 interconnected predicate claims of equivalence from 400 other devices. The vast majority of these devices (97%) are descended from only six surgical meshes that were present on the market prior to 1976. One of these ancestral meshes alone, provided the basis of 183 subsequent devices. Furthermore, we show that 16% of recently cleared devices are connected through equivalence claims to the 3 predicate meshes that have been recalled for design and material related flaws causing serious adverse events. Taken together, our results show that surgical meshes are connected through a tangled web of equivalency claims and many meshes recently cleared by the FDA have connections through chains of equivalency to devices which have been recalled from the market due to concerns over clinical safety. These findings raise concerns over the efficacy of the 510(k) route in ensuring patient safety.

## Introduction

Since 2008 and following an escalation in adverse events reported to the Manufacturers and the User Facility Device Experience (MAUDE) database, the US Food and Drug Administration (FDA) has issued a number of notifications associated with the long term and irreversible effects of using some of the surgical meshes available on the market [1,2,3]. All surgical meshes on the US market are designated by the FDA as potentially posing a moderate risk to patient health (class II) and were cleared through the 510(k) framework. Clearance through the 510(k) route is based on demonstrating the “substantial equivalence” of a device to an existing and previously cleared predicate device. Independent clinical evidence for the safety and effectiveness of the new device is rarely required [4,5,6,7,8]. The serious complications suffered by some patients have resulted in a number of class-action lawsuits and criticism that the 510(k) is not providing sufficient protection for patients [9,10,11,12]. In 2014, as a consequence of significant increases in the number of reported adverse events associated with the use of surgical mesh for pelvic floor repair, the FDA issued orders to reclassify two of the 37 groups of surgical meshes (synthetic and non-synthetic meshes for Pelvic Organ Prolapse repair) from class II to class III. As a result of this action manufacturers of new meshes as well as those currently on the market must submit a Pre-market Approval before those devices can be approved for marketing.

One of the key problems previously identified with the 510(k) framework is that despite the legal requirement that the scientific evidence demonstrating substantial equivalence to a predicate device be made publicly available, this information is often either lacking or insufficient [13]. However, even when substantial equivalence to a predicate is demonstrated, this does not ensure the safety of the device, if—as is exclusively the case—the predicates used were also cleared without evidence of safety and effectiveness [13]. Indeed, given repeated claims of substantial equivalency, across multiple generations of devices, the ultimate predicates of any new surgical mesh can eventually be traced back to devices that were marketed prior to the 1976 Medical Device Amendment Act. Prior to this act, regulation was limited to issues of misbranding and the hygiene of the manufacturing process and there was no additional requirement to prove the safety and efficacy of these devices [13]. Furthermore, given the requirement for substantial equivalence between devices, unrecognized design flaws present in a predicate have the potential to be passed on to any descendant devices inheriting these features. And yet, because the full genealogy of ancestral relationships connecting devices cleared through the 510(k) framework is not reported (only the immediate predicates of a device are listed), these inherited design flaws may often go undetected. As a result, even if a device has been recalled due to serious design flaws resulting in adverse patient outcomes, it is possible that numerous descendent devices, potentially sharing these critical flaws, may still be on the market and in regular clinical use. The inherited design flaws that go undetected in such cases have the potential to cause serious complications in patients receiving these devices but the extent of this problem remains unclear.

Here we use the FDA 510(k) database to investigate the ancestral pathway through which surgical meshes have come to market. Given the large number of devices involved we employ a network approach that enables us to rapidly trace the connections between any selected device and both its predicates and descendants. Using this technique, we address three key aims. First, we determine the number of predicates on which each device is based and how this accumulates over multiple generations of substantial equivalency claims. Second, we determine the number of meshes that are descended from predicates that have since been recalled for design related flaws. Finally, we evaluate the potential risk of inheriting design related flaws by quantifying the number of original meshes from which all recently cleared devices have descended and the degree of interrelatedness amongst these meshes. For those meshes that have resulted in the highest number of descendants we critically examine the original scientific and/or clinical evidence for their safety and effectiveness.

## Methods

We identified all new devices under the product code ‘surgical mesh’ cleared through the 510(k) framework between the 1^st^ of January 2013 and the 31^st^ of December 2015 (n = 77) and traced their ancestry through multiple generations of predicates (S1 Data). For each device, we identified the predicates that were used as substantial equivalents, and then repeated this process backwards through time for as many generations as possible given the information available on the FDA website (https://www.fda.gov). In some cases, the identity of the predicates was not provided preventing a search into the deeper ancestry of that device.

Tracing the ancestry of devices is time consuming, because this information has to be extracted from the individual 510(k) device summaries. A previous study by Zuckerman et al [13] examined the evidence for substantial equivalence claims, across a variety of implantable devices categories (cardiovascular, dental, general and plastic surgery, neurological and orthopedic) but focused on reconstructing the ancestry of two cleared devices per category per year, from 2008 to 2012, thus including a total of ten surgical meshes. In contrast, here we perform a more in-depth analysis of all 77 surgical meshes cleared between 2013–15 and their ancestral predicates. In total, we examined 771 device-predicate connections across 477 unique surgical meshes.

We recorded the years in which each predicate device was cleared for market, whether that device was cleared prior to the 1976 Medical Device Amendment Act and whether the device had subsequently been recalled. The FDA uses the term “recall” when a manufacturer takes a correction or removal action to address a problem with a medical device that violates laws administered by the FDA. According to the FDA website “Recalls occur when a medical device is defective, when it could be a risk to health, or when it is both defective and a risk to health”. In most cases, a company (manufacturer or distributor) voluntarily recalls a medical device that is in violation of the FDA laws by notifying the FDA of the potential problems and initiating the recall through correction or removal of the device from the market. However, in cases where the company fails to recall the defective device, the FDA has the legal right to recall the device based on the evidence of a potential risk to public health. The FDA then reviews and monitors the recall strategy of the recalling firm. In the case of recalled implantable devices, such as meshes, which have the potential to fail unexpectedly, companies often request the relevant medical professionals to contact the patients and discuss the risk of removing the device compared to the risk of leaving it in the body. Examples of the types of actions that may be considered for a recalled device include: inspecting the device for potential problems, monitoring patients for health issues and in some cases removing the implant from the body. Devices may be recalled for numerous reasons including those not directly associated with the device in question (e.g. issues with packaging or sterilisation). For each recalled device we therefore searched for the cause of the recall from the FDA medical recalls database. We identified those devices recalled due to ‘design and material related flaws’ (e.g. mechanical problems leading to device breakage or failure).

To investigate the historical patterns of ancestry between recently cleared devices we generated a network describing the ancestor-descendent relationships between all 477 meshes. This network is akin to a genealogical tree. Each node represents a unique mesh with the links between nodes indicating when a device was used as a predicate or ‘parent’ of another device. The terminal nodes of the tree correspond to devices that have yet to be used as a predicate of another device. We first used this network to take a ‘bottom-up’ approach (i.e. from the present to the past), quantifying for each mesh both the number of first-generation predicates on which substantial equivalency was based and the total number of predicates in the meshes ancestry, potentially stretching back over many generations. We quantified the total number of predicate devices cleared during each year since 1976 and the number of generations connecting each device to its founder device (i.e. the earliest device listed on the 510(k) database). We calculated the proportion of meshes with a device in their ancestry that i) was placed on the market prior to the 1976 Medical Device Regulation Act or ii) had been recalled for ‘design and material related flaws’.

We then took a ‘top-down’ approach, and for each mesh in our dataset calculated the number of devices descending from that mesh. For those meshes with more than 100 descendants we used the device summaries to record whether clinical or pre-clinical evidence was provided and if so the type of evidence used.

All analyses were conducted in the R statistical programming environment using the ‘graph’ and ‘igraph’ libraries.

## Results

**Fig 1:**
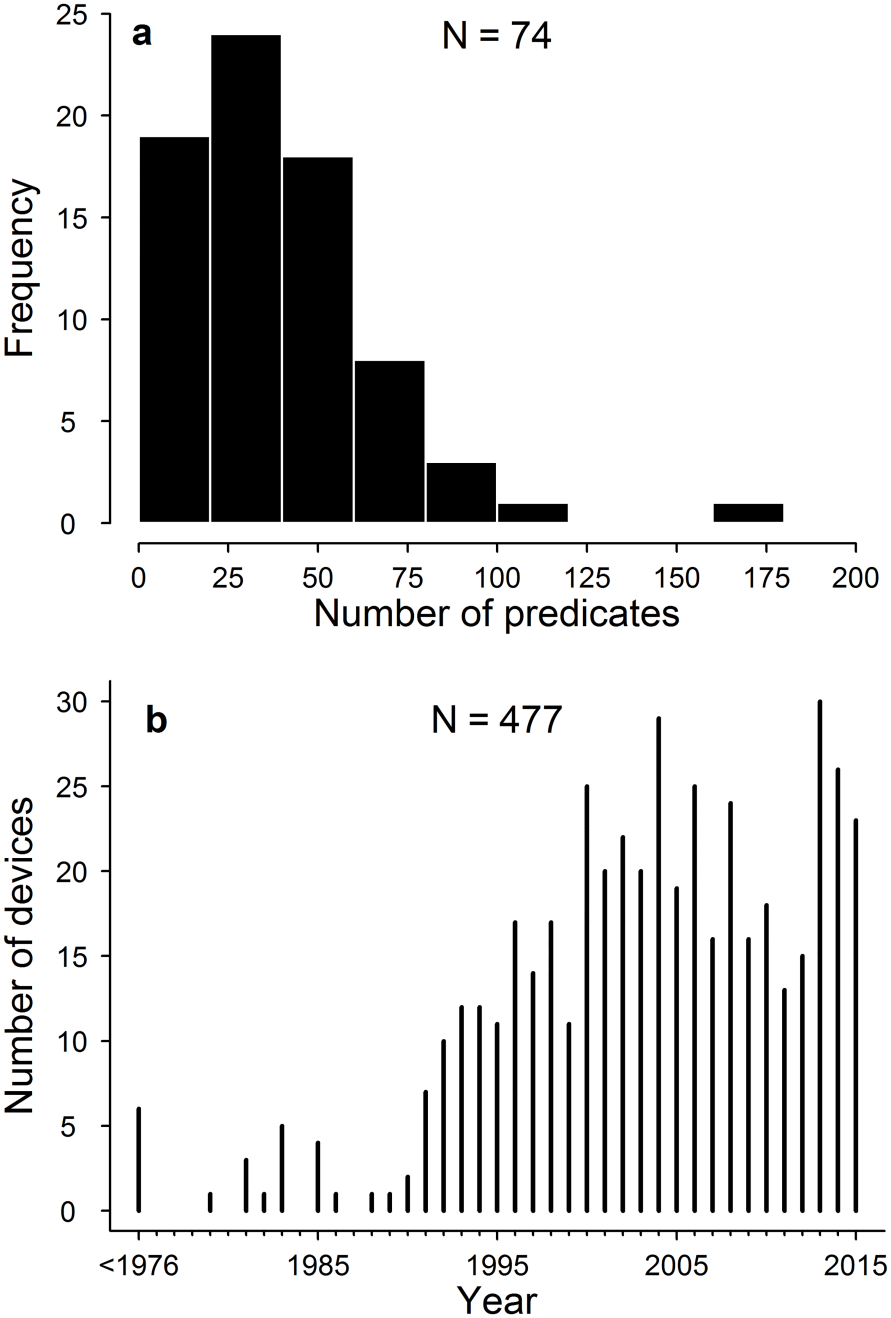
a) The number of ancestral predicates underlying surgical meshes cleared by the FDA between 2013–2015. Each surgical mesh has on average 33 ancestral predicates, but the number of ancestors differs widely between meshes b) the number of devices in our dataset (n = 477) cleared by the FDA each year from prior to the 1976 Medical Device Amendment Act 2015 (i.e. < 1976) to 2015.

Of the 77 surgical meshes cleared in the study period, 74 provided information on the identity of the predicates on which substantial equivalence was based. The number of predicates cited by each mesh varied from one to seven, but on average only a single predicate was used (median = 1, mean = 2). However, because the predicates of each device were themselves cleared through the 510(k) framework the total number of ancestral predicates underlying a device is substantially greater (Fig 1a). The median total number of unique predicates accumulated by each device was 33 (mean = 39), spread across, on average, seven generations. The maximum number of ancestral predicates for any mesh was 165 (device 510(k) number = “K133223”) (Fig 1a). In total we identified 771 predicate devices from which the 77 devices had arisen. This is an underestimate of the true number of predicates because in 128 cases no information on the identity of the predicates was available, either because this was not listed in the summary sheet (n = 22) or because the summary sheet was not provided (n = 106). Of the 771 predicates, only 400 of these represent unique devices because many devices share the same predicates.

Of the 74 recently cleared meshes for which information on their ancestry was available, the predicates of 52 meshes (70%) could be traced back to surgical meshes arising prior to the 1976 Medical Device Amendment Act. In all other cases, ancestral relationships were terminated by devices for which no information on predicates was provided. Had this information been available, all surgical meshes cleared between 2013 and 2015 would have ultimately arisen from a pre-amendment device. Meshes cleared between 2013 and 2015 rarely directly cite pre-amendment devices (n = 1), but in many cases it was possible to trace a mesh back to a pre-amendment device within only two (n = 9, 17%) or three (n = 19, 37%) generations. Thus, despite the fact that pre-amendment devices were not subject to safety and effectiveness standards, they continue to provide the basis of substantial equivalence for surgical meshes currently entering the market.

Across the 74 surgical meshes recently cleared, the average date at which their immediate predicates entered the market was 2009. In other words, most devices typically cite predicates that entered the market on average only 4.5 years earlier. Although each mesh necessarily has at least one immediate predicate, the total number of ancestral predicate devices decreases with time before the present because many devices are used in multiple equivalency claims (Fig 1b). For instance, all of the 74 new devices stem from only 23 predicates that entered the market prior to 1990 and these in turn are ultimately derived from only six pre-amendment devices (Fig 1b). This pattern, whereby all devices ultimately coalesce into a small number of ancestral predicates is partially explained by the absence of information on the predicates for some devices. However, this is not the main driver of this trend, which instead simply reflects the fact that fewer devices were available on the market in the past [14].

**Fig 2:**
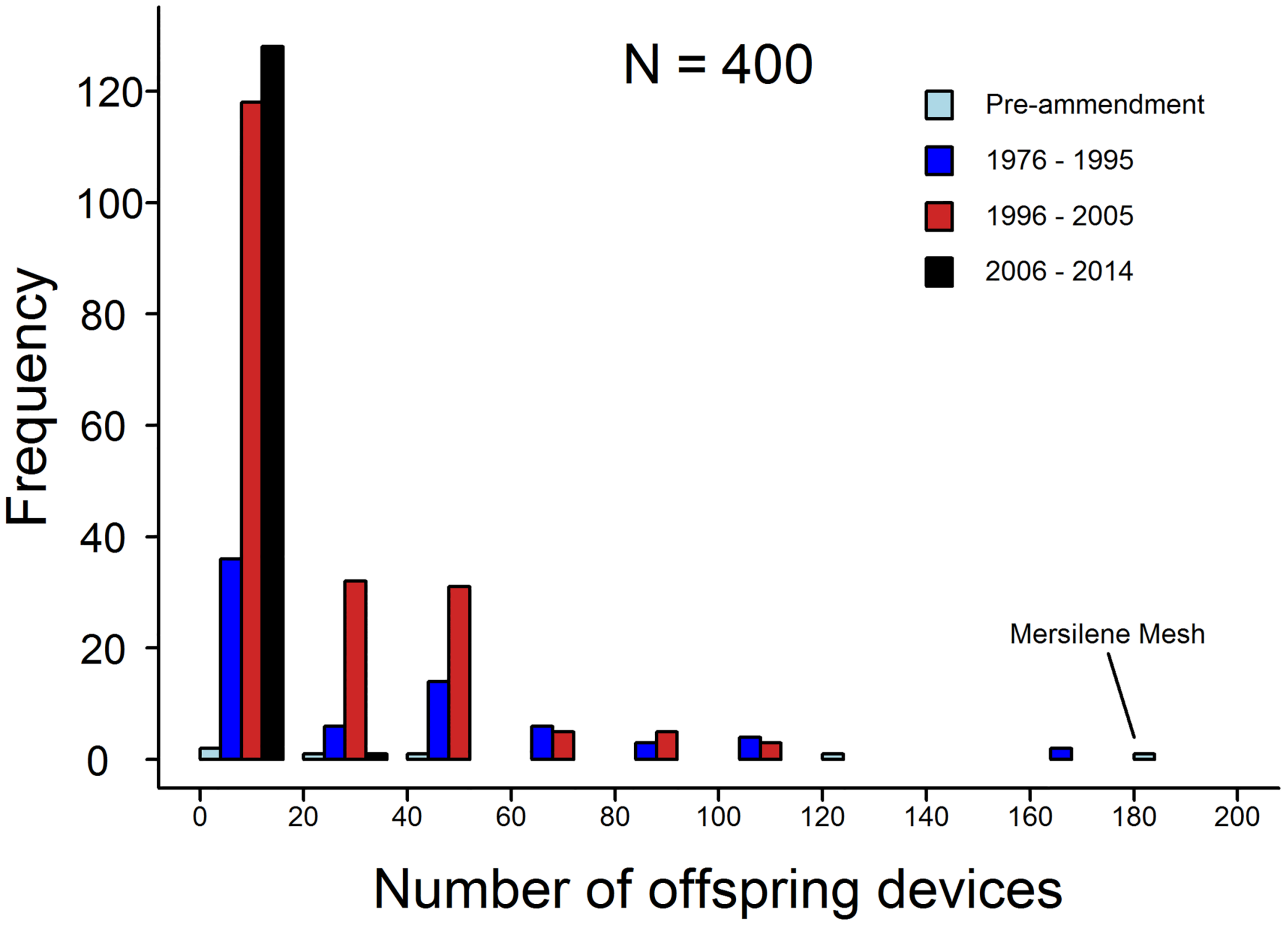
The total number of descendent devices connected to each ancestral predicate (n = 400) by chains of substantial equivalency. Ancestral predicates are grouped according to the time period in which they entered the market (bar color) to highlight that the skewed distribution in the number of descendent devices is not an artefact of the time available for ancestral predicates to accumulate descendants. Mersilene Surgical Mesh had the largest number of descendent devices and is highlighted (see Fig 3 for the ancestral history of this device).

**Fig 3:**
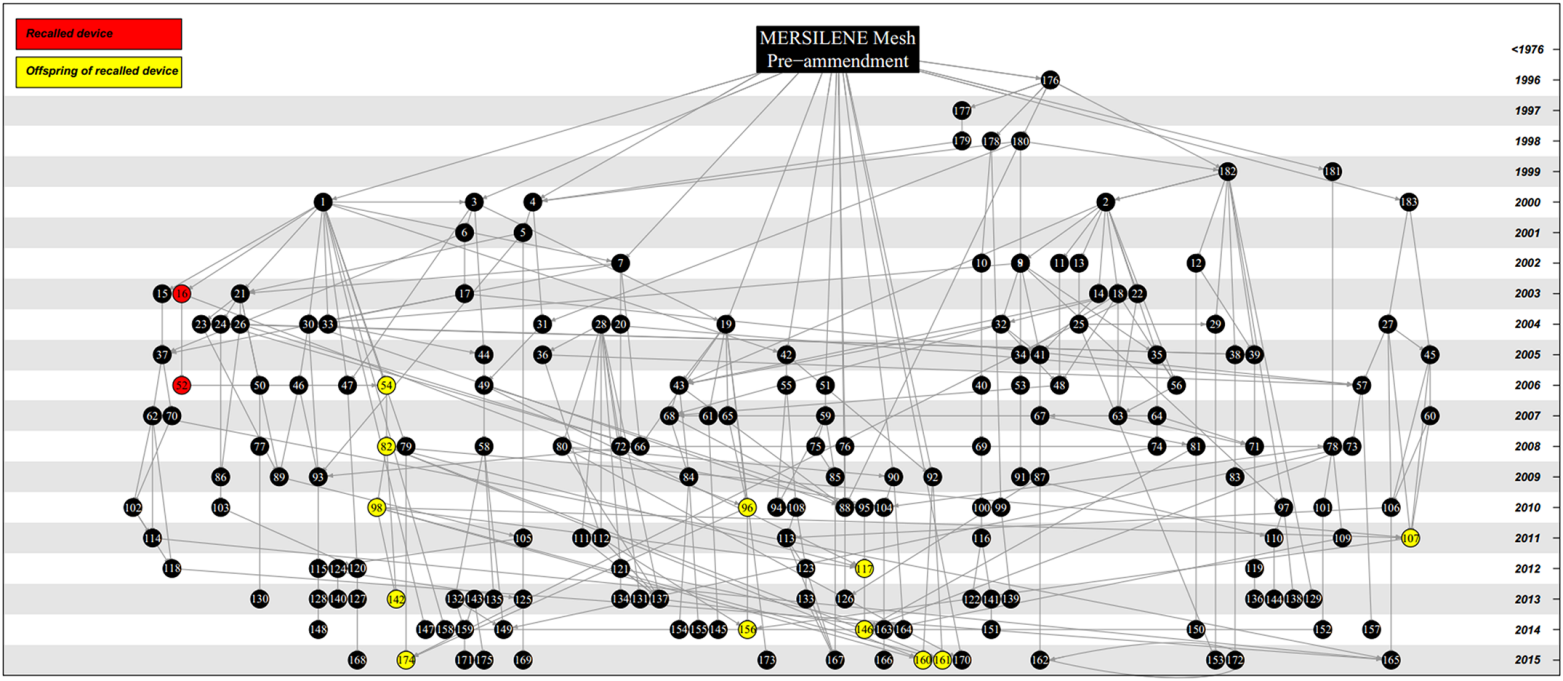
The ancestral device network of Mersilene Surgical Mesh manufactured by Ethicon Inc. Mersilene Mesh has led to 183 descendent devices. Devices in the ancestral network that have since been recalled for ‘design and material related flaws’ (n = 2) are highlighted in red. Devices that are descended from recalled devices by substantial equivalency chains (n = 12) are highlighted in yellow (see S2 Data for devices).

We found that the number of descendent devices derived from each predicate is highly skewed. For instance, while some devices have only been used as a predicate once, we identified twelve devices that have each ultimately led to over 100 descendants (Fig 2). This variation is not simply an artifact of some predicate devices entering the market earlier than others and thus having longer to accumulate descendent devices, because this pattern is also present amongst devices of similar age (Fig 2). For instance, the six pre-amendment devices in our dataset have collectively led to 387 descendent devices, but 303 (78%) of these are derived from just two pre-amendment devices, Mersilene Mesh, which has 183 descendants (Fig 3), and Prolene Polypropylene Mesh, which has 120 descendants.

The lack of available scientific evidence to support claims of substantial equivalence has previously been noted by Zuckerman et al [13] who used a similar methodology to assemble information on the predicate history of implants from the FDA website. To explore this further, we analysed the scientific and clinical data publicly available in the 510(k) summaries of those meshes with more than 100 descendants (n = 11). Of these 11 devices, two are pre-amendment devices (Mersilene Mesh and Prolene Polypropylene Mesh) and thus no information about their safety and efficacy is available on the FDA website. Despite the legal requirements, summaries for six devices (50%) are absent from the FDA database [15]. Finally, of the three remaining devices, all claim to have performed some bench testing but no details of the tests or results are available and, in all cases, no clinical data is presented (Table 1).

**Table 1:** Public availability of clinical and scientific evidence for the meshes that have led to over 100 off-spring devices.

Device Name	Manufacturer	510K Number	Date cleared	Summary	Evidence mentioned / Data Provided	Number of descendants
**Mersilene Mesh**	Ethicon INC	-	Pre 1976	-	None	183
**SUPPLE PERI-GUARD**	BIO-VASCULAR, INC.	K923657	12/21/1992	No summary available	None	167
**BARD MARLEX MESH DART**	C.R. Bard, INC	K922916	08/24/1992	No Summary available	None	162
**PROLENE PROPROPYLENE MESH NONABSORBABLE SYNTHETIC SURGICAL MESH**	ETHICON, INC.	-	Pre 1976	-	None	120
**MODIFIED PROLENE POLYPROPYLENE MESH NONABSORBABLE SYNTHETIC SURGICAL MESH**	ETHICON, INC.	K962530	08/09/1996	Summary available	No clinical evidence Bench testing: Only Burst strength test	119
**DEXON 'S' POLYGLYCOLIC ACID MESH**	Davis & Geck, INC	K830889	05/09/1983	No Summary available	None	118
**PROLENE SOFT (POLYPROPYLENE), NONABSORBABALE SYNTHETIC SURGICAL MESH**	ETHICON, INC.	K001122	05/23/2000	Summary available	No clinical evidence Nonclinical laboratory testing was not performed According to the manufacturer bench testing was performed but the details were not provided	115
**TISSUE PATCH, TISSUE GRAFT**	BIO-VASCULAR, INC.	K921895	11/04/1992	Statement available	None	106
**TRELEX NATURAL(R) MESH**	MEADOX MEDICALS, DIV. BOSTON SCIENTIFIC CORP.	K945377	12/08/1994	No summary available	None	104
**GORE-TEX SOFT TISSUE PATCH,SURGICAL MEMBRANE,MESH**	W.L. GORE & ASSOCIATES,INC	K930822	01/28/1994	No summary available	None	104
**SURGICAL FABRICS**	BOSTON SCIENTIFIC CORP.	K963226	11/15/1996	Summary available	No clinical evidence According to the manufacturer bench testing was performed but details were not provided	103

**Fig 4:**
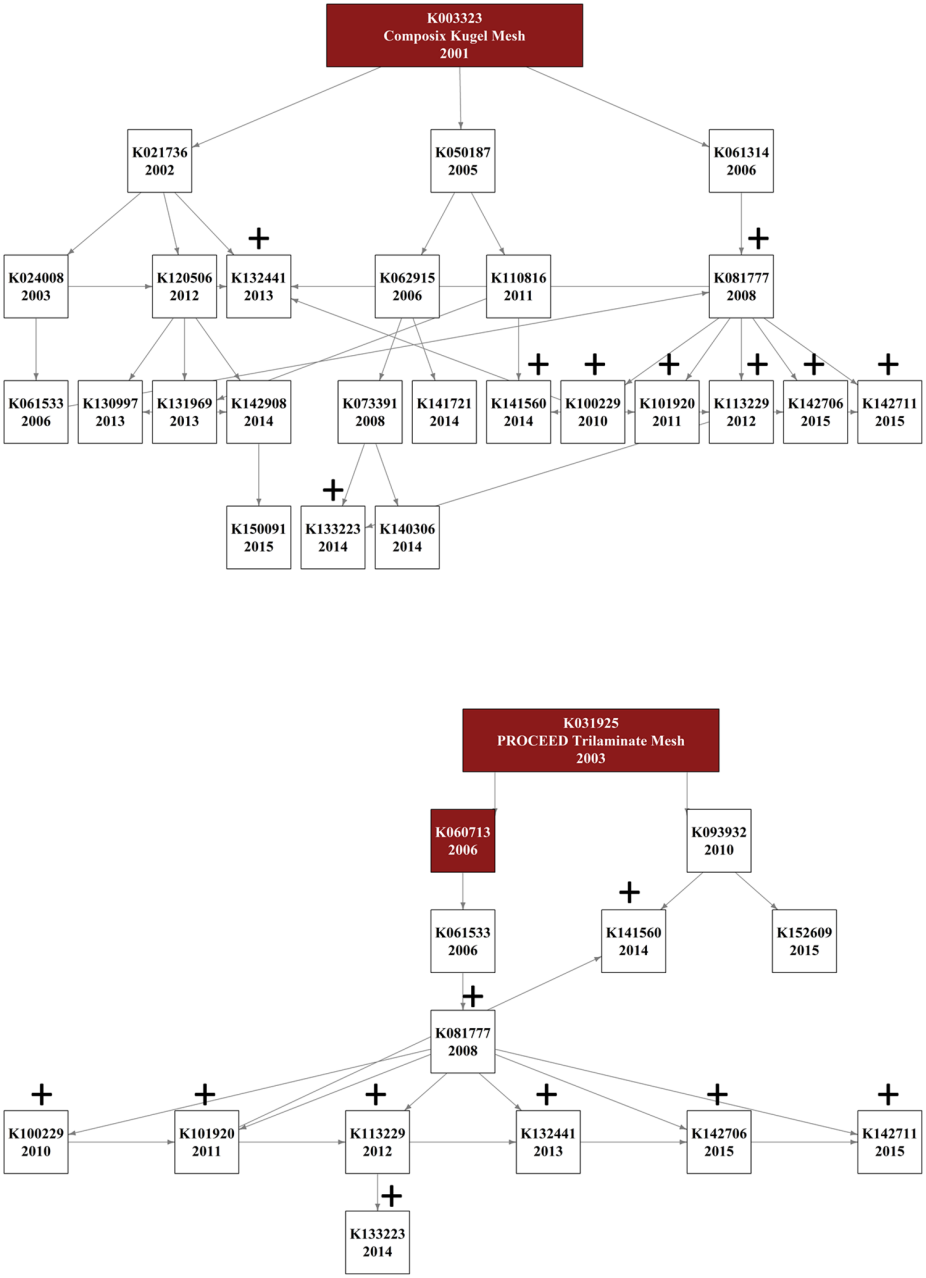
The ancestral device network of the recalled devices Composix Kugel Mesh (K003323) and PROCEED Trilaminate SurgicalMesh (K031925). **+** Shows devices with more than 2 recalled predicates.

To assess the potential risks associated with the non-independent ancestry of devices we calculated the number of recently cleared devices that have been derived from predicates that have since been recalled. Across our dataset of 400 unique predicate devices, we identified three devices that had been recalled due to design and material related flaws that resulted in serious long term and irreversible adverse events (Composix Kugel Mesh (K003323), PROCEED Trilaminate SurgicalMesh (K031925) and PROCEED Surgical Mesh (K060713)) (Fig 4). These recalled meshes represent less than 1% of all the devices in our dataset. However, because these recalled meshes were on the market for a period of up to 5 years before being recalled (K003323, 5 years; K031925, 3 years; K060713, 5 years) they served as predicates for multiple descendent meshes (K003323, 24 descendent devices; K031925, 13 descendent devices; K060713, 10 descendent devices). In fact, we found that 12 out of the 74 (16%) devices cleared between 2013 and 2015 have descended from one of these three recalled devices, with five surgical meshes containing all three recalled devices in their ancestral tree. Furthermore, these recalled devices are often very closely related to those recently entering the market: on average one only has to travel back three generations in the predicate ancestry to reach a device that has since been recalled.

## Discussion

Surgical meshes have been in use for over sixty years for a wide variety of soft tissue repair indications. Many patients have benefited from the implantation of these devices. Tragically, a significant number of patients have also suffered from long term and irreversible complications related to surgical meshes. All surgical meshes that have entered the market have done so through the 510(k) process, in which new devices are cleared simply if they provide evidence of substantial equivalence to at least one predicate device already on the market. Although it is important to acknowledge that some of the complications experienced by patients may have arisen from poor surgical techniques or the general risks associated with surgical procedures, the significant number of adverse cases associated with serious design flaws and complications related to the type of materials used in surgical meshes remains a concern [9,12,13].

The key underlying assumption of the 510(k) framework is that if a new mesh is sufficiently similar to a previous device, then it will be at least as safe and effective as the devices currently on the market. However, in the vast majority of cases, the devices used as predicates in claims of equivalence have never been adequately assessed for safety and effectiveness [13,16], raising the possibility that undetected design related flaws present in a device may inadvertently be passed on to multiple further devices. Because claims of substantial equivalence are often loosely defined, the degree which design-related flaws are inherited from predicate to descendent devices may be difficult to quantify. However, our analysis reveals the potential extent of this problem, by showing that the substantial equivalence principle used in the 510(k) route has resulted in an extremely high degree of connectedness amongst surgical meshes. Almost all new surgical meshes are ultimately derived from only a handful of ancestral devices that lack clinical data and sufficient scientific evidence.

Across surgical meshes, we found that direct claims of equivalency were often based on predicates that had been on the market for only a few years. This short ‘generation time’ between ancestral and descendent devices, raises the possibility that by the time a device has been recalled due to safety issues, many substantially equivalent devices sharing these problems may have since entered the market. Our results, highlight the magnitude of this risk by demonstrating that even though the number of recalled surgical meshes is relatively small, a substantial proportion of meshes recently entering the US market are closely connected to these recalled meshes through claims of substantial equivalence. We emphasize that by only focusing on recalled devices our analysis provides a conservative estimate of the number of devices potentially causing health related problems because even when such design and material related flaws have been identified, and the use of a device has been discontinued, this rarely results in a recall by the FDA.

Furthermore, although most devices cite only one or two predicates, the total number of predicates underlying a device is substantially greater than this and most devices are the product of a long chain of substantial equivalence claims stretching back decades. This process can result in new devices entering the market that are substantially dissimilar to their predicates, a phenomenon known as ‘predicate creep’ [17]. An example of this problem was the clearance of metal on metal hip implants and the ReGen Menaflex collagen scaffold where small differences between a device and its predicate were magnified over time and among the offspring predicate devices [17,18,19,20]. This general issue has been identified previously [13], but our in-depth analysis of all surgical meshes cleared from 2013–2015, further highlights the lack of publicly available scientific evidence to support claims of substantial equivalence, safety, or effectiveness in this group of surgical implants. These results identify concerns with the 510(k) framework and we therefore suggest that the clearance of certain groups of class II devices, such as surgical meshes that have the potential to cause irreversible complications, should be supported by clinical data and rigorous pre-clinical evaluations, rather than simply claims of equivalence to pre-existing devices. In 2014, as a consequence of major complications reported in the medical literature and a series of legal claims for compensation by patients harmed by certain types of pelvic floor repair meshes, the FDA issued orders to reclassify two of the 37 groups of surgical meshes (synthetic and non-synthetic meshes for Pelvic Organ Prolapse repair) from class II to class III. This change required manufacturers to submit a pre-market approval (PMA) application. However, to date, no manufacturer has chosen to do this, and thus effectively all surgical meshes currently on the US market continue to be cleared through the 510(k) route.

The first step towards achieving a suitable regulatory system would be to define appropriate clinical trial designs for first-in-man studies and a standardized reporting system to uniformly assess the safety and effectiveness of devices based on their potential risk to the human body. Another important parallel improvement is to develop a uniform set of requirements based on international standards for reporting the scientific data and pre-clinical tests performed by manufacturers and to ensure that the information is available for public scrutiny. Finally, the FDA currently only provides information on the immediate predicates used by a device and thus uncovering the deeper ancestral relationship between devices is extremely time consuming. If the FDA were to publish the full genealogical relationships between devices on their website this would enable researchers to rapidly check whether any of the devices they have used as predicates have descendent from other devices for which flaws have since been identified. We argue that this would represent a simple but highly effective way of minimizing the risk that design or material related flaws causing adverse patient outcomes are propagated amongst devices.

## Conclusion

The framework for the current regulatory system for approval and clearance of medical devices was created 40 years ago in an era of much simpler medical technologies and a smaller number of devices. Recent complications with a number of surgical meshes and other medical devices have raised questions regarding the extent to which the current 510(k) process is capable of safeguarding public health and our study identifies a number of further concerns over the efficacy of this regulatory framework. We believe that the 510(k) framework—based on the use of multiple predicates and claims of substantial equivalence to predicate devices with no clinical data and limited pre-clinical testing—is not fit for purpose and should be discontinued. We suggest that a more forward looking and evidence based regulatory framework with the capacity to efficiently analyze scientific and clinical data for each implantable Class II device is urgently required.

## Supporting information

S1 Data Devices cleared. Table of surgical meshes cleared by the FDA between 2013–2015, including details of the manufacturer and their unique 510(k) number.
(PDF)

S2 Data Mersolene daughters. Table providing the 510(k) numbers of surgical meshes in the ancestral device network in Fig 3.
(XLSX)
