# Supplementary material for: The regulatory ancestral network of surgical meshes
Source: PLoS One. 2018 Jun 19;13(6):e0197883. doi: 10.1371/journal.pone.0197883 (PMC6007828; doi:10.1371/journal.pone.0197883)
Supplement: S1 Data — Table of surgical meshes cleared by the FDA between 2013–2015, including details of the manufacturer and their unique 510(k) number. (PDF) [file pone.0197883.s001.pdf]

Name	Manufacturer	knumber	Date
DESARA SL	CALDERA MEDICAL INC.	K121928	2013
COLLAGEN TENDON SHEET-D	ROTATION MEDICAL INC.	K122048	2013
VERTESSA LITE 10 X20CM VERTESSA LITE 11 X 30CM VERTES	CALDERA MEDICAL INC.	K123028	2013
SERI SURGICAL SCAFFOLD	ALLERGAN	K123128	2013
VERTESSA LITE 10 X 20CM VERTESSA LITE 11 X 30CM	CALDERA MEDICAL	K123337	2013
FORTIVA PORCINE DERMIS	RTI BIOLOGICS INC.	K123356	2013
RESTORELLE Y CONTOUR	COLOPLAST A/S	K123914	2013
BIODESIGN SURGISIS ANTERIOR POSTERIOR PELVIC FLOOR (	COOK BIOTECH INCORPORATED	K130006	2013
ENDOFAST RELIANT SCP ENDOFAST RELIANT LAP	IBI ISRAEL BIOMEDICAL INNOVATIONS LTD.	K130059	2013
COVAMESH	BIOMUP S.A.	K130428	2013
ENDOFORM RECONSTRUCTIVE TEMPLATE	MESYNTHE LTD	K130547	2013
LTN - LAPAROSCOPIC SURGICAL MESH	LIFECELL CORP.	K130817	2013
AIGIS RX R PM/ AIGIS RX R ICD	TYRX INC.	K130943	2013
VENTRALIGHT ST MESH WITH ECHO PS POSITIONING SYSTE	C.R. BARD INC.	K130968	2013
NEOVEIL	GUNZE LIMITED	K130997	2013
AIGIS RX N MEDIUM; AIGIS RX N LARGE	TYRX INC	K131007	2013
TRELLIS COLLAGEN RIBBON	WRIGHT MEDICAL TECHNOLOGY INC.	K131143	2013
SPARC SYSTEM AND MONARC MONARC AND MONARC C SY	AMERICAN MEDICAL SYSTEMS	K131229	2013
DYNAMESH -CICAT	FEG TEXTILTECHNIK FORSCHUNGS-UND ENTWICKLUNGSGESEL	K131530	2013
GORE SEAMGUARD REINFORCEMENT	W. L. GORE & ASSOCIATES INC.	K131658	2013
SYMBOTEX(TM) COMPOSITE MESH	SOFRADIM PRODUCTION	K131969	2013
MESO BILAYER SURGICAL MESH	KENSEY NASH CORPORATION DBA DSM BIOMEDICAL	K132025	2013
GYNECARE TVT EXACT CONTINENCE SYSTEM	ETHICON INC.	K132054	2013
RESTORELLE M RESTORELLE XL	COLOPLAST CORP.	K132061	2013
DESARA MESH DESARA BLUE	CALDERA MEDICAL INC.	K132069	2013
BARD VENTRALEX HERNIA PATCH	C.R. BARD INC.	K132441	2013
RETROARC RETROPUBIC SLING SYSTEM	AMERICAN MEDICAL SYSTEMS INC.	K132655	2013
AIGIS RX N (MEDIUM) AIGIS RX N (LARGE)	TYRX INC.	K132699	2013
MESO TENDON MATRIX	KENSEY NASH CORPORATION DBA DSM BIOMEDICAL	K133169	2013
AVANCEÂ® FOAM ABDOMINAL DRESSING KIT	MOLNLYCKE HEALTHCARE	K130852	2014
DIAPHRAGMATIC HERNIA GRAFT	COOK BIOTECH INC.	K133011	2014
ULTRAPRO COMFORT PLUG	ETHICON INC.	K133198	2014
XENMATRIX AB SURGICAL GRAFT	C.R. BARD INC.	K133223	2014
SIS HERNIA GRAFT	COOK BIOTECH INC.	K133306	2014
PREMIUM	COUSIN BIOTECH S.A.S.	K133889	2014
MIROMATRIX BIOLOGICAL MESH	MIROMATRIX MEDICAL INC.	K134033	2014
RESTORELLE Y CONTOUR MESH	COLOPLAST A/S	K140116	2014
COLLAGEN TENDON SHEET-DDI (CTS-DDI)	ROTATION MEDICAL INC.	K140300	2014
CORMATRIX PROTECT ECM ENVELOPE	CORMATRIX CARDIOVASCULAR INC.	K140306	2014
XENMATRIX SURGICAL GRAFT	C.R. BARD	K140501	2014
DESARA BLUE OV DESARA BLUE SS	CALDERA MEDICAL	K140843	2014
PROGRIP SELF-GRIPPING POLYPROPYLENE MESH PARIETENI	SOFRADIM PRODUCTION	K140941	2014
FREEDOM INGUINAL HERNIA REPAIR IMPLANT-25MM/40M	Insightra Medical	K140967	2014
OVINE TISSUE MATRIX(OTM)	TELA BIO INC.	K141053	2014
X-REPAIR	SYNTHASOME	K141394	2014
X-REPAIRSL	SYNTHASOME INC.	K141499	2014

ETHICON PHYSIOMESH OPEN FLEXIBLE COMPOSITE MESH D	ETHICON INC.	K141560	2014
BOVINE PERICARDIUM PATCH	COLLAFIRM LLC	K141721	2014
FORTIVA PORCINE DERMIS TUTOPLAST PORCINE DERMIS	RTI SURGICAL INC.	K142070	2014
PARIETENE MACROPOROUS MESH	SOFRADIM PRODUCTION	K142091	2014
HP Tissue Matrix	LifeCell Corporation	K142326	2014
TYRX Neuro Absorbable Antibacterial Envelope	MEDTRONIC TYRX INC.	K142611	2014
Polyester Mesh)<br>Parietex Plug and Patch (new name : Parietex Plug and Patch System)<br>Progrip Laparoscopic Self-Fixating Mesh	SOFRADIM PRODUCTION	K142900	2014
Parietex Lightweight Mesh)<br>Parietex Composite Mono PM Mesh (new name: Parietex Composite Parastomal Mesh)<br>Parietex Composite Ventral Patch Symbotex Composite Mesh	SOFRADIM PRODUCTION	K142908	2014
Matristem Surgery Matrix RS PSM PSMX Matristem Pelvic Fl	ACELL INC	K141084	2015
Insightra Freedom Ventral Hernia Repair System	Insightra Medical	K142192	2015
Modified ONFLEX Mesh	C.R. Bard Inc.	K142706	2015
ONFLEX Mesh	C.R. Bard Inc.	K142711	2015
SIS Inguinal Hernia Repair Graft	COOK BIOTECH INCORPRATED	K142887	2015
Device<br>RENASYSÂ¿ AB Abdominal Dressing Kit with Soft Port	SMITH & NEPHEW INC.	K143133	2015
Phasix ST Mesh	C. R. Bard Inc	K143380	2015
Progrip Laparoscopic Self-Fixating Mesh	SOFRADIM PRODUCTION	K143386	2015
ECHO 2.0 LAP SYSTEM with VENTRALIGHT ST MESH	C.R. Bard Inc.	K143743	2015
Vertessa Lite	CALDERA MEDICAL INC.	K150016	2015
Vertessa Lite Y-Mesh	CALDERA MEDICAL INC.	K150023	2015
Versatex Monofilament Mesh	SOFRADIM PRODUCTION	K150091	2015
TYRX Neuro Absorbable Antibacterial Envelope	MEDTRONIC TYRX INC.	K150291	2015
Miromatrix Biological Mesh RS	MIROMATRIX MEDICAL INC.	K150341	2015
GORE SEAMGUARD Reinforcement	W. L. GORE & ASSOCIATES INC.	K150551	2015
Biodesign Enterocutaneous Fistula Plug	COOK BIOTECH INCORPRATED	K150668	2015
LTM-Perforated Surgical Mesh	LifeCell Corporation	K150712	2015
ULTRAPRO ADVANCED Macroporous Partically Absorbable M	EHICON INC.	K150906	2015
XenMatrix AB Surgical Graft	C.R. Bard	K151177	2015
C-QUR C-QUR FX C-QUR TacShield C-QUR V-Patch C-QUR Ce	ATRIUM MEDICAL CORPORATION	K151386	2015
ProLite Mesh ProLite Ultra Mesh ProLoop Mesh Plug	ATRIUM MEDICAL CORPORATION	K151437	2015
GORE SYNECOR Biomaterial	W.L GORE & ASSOCIATES INC.	K152609	2015
TYRX Neuro Antibacterial Envelope	TYRX INC.	K152678	2015
